# Correction to: The impact of the COVID-19 pandemic and associated public health response on people with eating disorder symptomatology: an Australian study

**DOI:** 10.1186/s40337-022-00549-2

**Published:** 2022-02-14

**Authors:** Jane Miskovic-Wheatley, Eyza Koreshe, Marcellinus Kim, Rachel Simeone, Sarah Maguire

**Affiliations:** 1 InsideOut Institute, Central Clinical School, Faculty of Medicine and Health, The University of Sydney, Charles Perkins Centre, Camperdown, NSW 2006 Australia; 2 Sydney Local Health District, St Leonards, NSW Australia

## Correction to: Journal of Eating Disorders (2022) 10:9 10.1186/s40337-021-00527-0

Following the publication of the original article [1], the authors brought to our attention that an error was unfortunately introduced to Table 3 during the implementation of their corrections by the correction team:

A graphic intended to be included in Table 3, inadvertently replaced the entire Table.

The correct Table 3 is shown here below and has now been included in the original article.

**Table 3:**
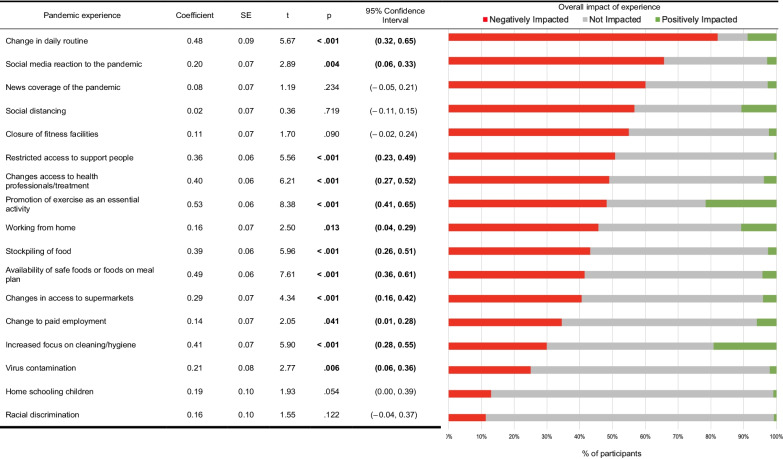
Pandemic experience and impact on eating disorder index (*N* = 1723)

N, sample size; SE, standard error; CI, confidence interval; *p*, *p*-value significant at *p* < .05; t*,* t-statistic
